# First-Principles Studies of the Electronic and Optical Properties of Two-Dimensional Arsenic–Phosphorus (2D As–P) Compounds

**DOI:** 10.1021/acsomega.4c04108

**Published:** 2024-08-10

**Authors:** Jose Mario Galicia Hernandez, Jonathan Guerrero-Sanchez, Jairo Arbey Rodriguez-Martinez, Noboru Takeuchi

**Affiliations:** † Centro de Nanociencias y Nanotecnología, Universidad Nacional Autónoma de México, Ensenada, Baja California 22860, Mexico; ‡ Departamento de Física, Universidad Nacional de Colombia, Bogotá DC 111311, Colombia

## Abstract

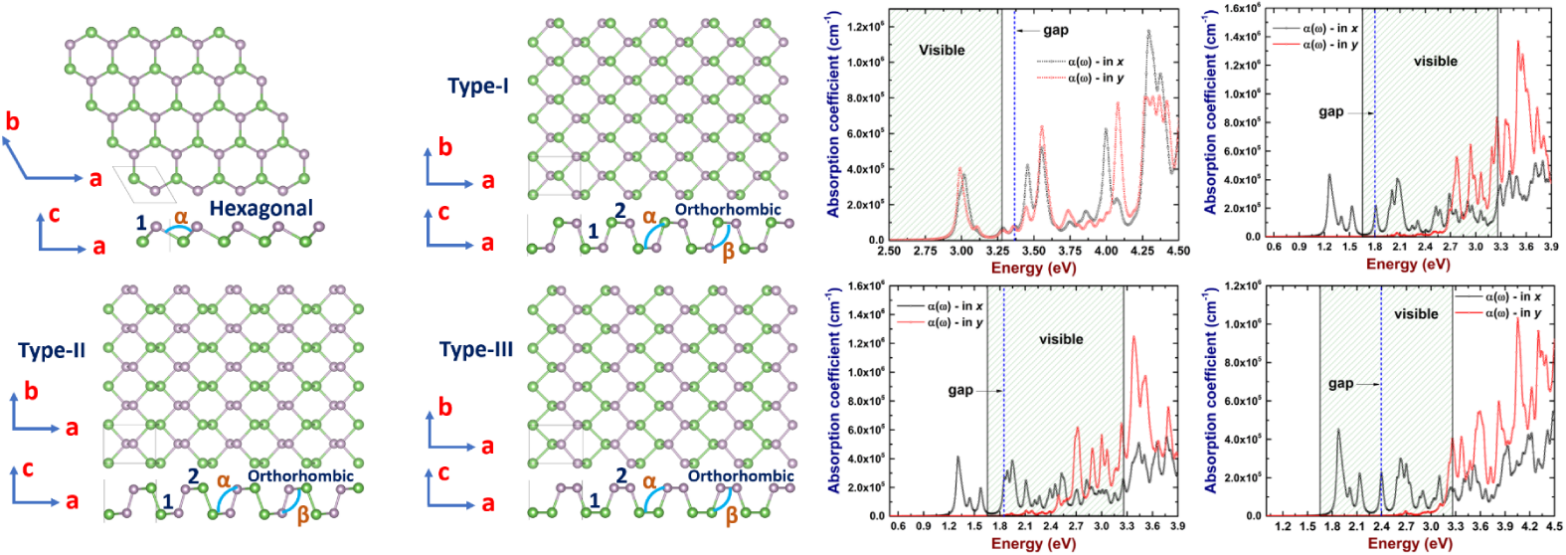

In this work, we propose the construction of a two-dimensional system based on the stable phases previously reported for the 2D arsenic and phosphorus compounds, with hexagonal and orthorhombic symmetries. Therefore, we have modeled one hexagonal and three possible orthorhombic structures. To ensure the dynamical stability, we performed phonon spectra calculations for each system. We found that all phases are dynamically stable. To ensure the thermodynamic and mechanical stabilities, we have calculated cohesive energies and elastic constants. Our results show that the criteria for the stabilities are all fulfilled. For these stable structures, we computed the electronic and optical properties from first-principles studies based on density functional theory. The computation of electronic band gaps was performed by using the GW approximation to overcome the underestimation of the results obtained from standard DFT approaches. To study the optical properties, we have computed the dielectric function imaginary part within the BSE approach, which takes into account the excitonic effects and allows us to calculate the exciton binding energies of each system. The study was complemented by the computation of the absorption coefficient. From our calculations, it can be established that the 2D As–P systems are good candidates for several technological applications.

## 1 Introduction

Since the discovery of graphene in 2004, there has been increasing interest in the study and development of new 2D materials with outstanding properties.^1−5^ Although graphene possesses important properties such as high conductivity,^6^ high carrier mobilities,^7^ excellent thermal conductivity,^8^ quantum confinement effect,^9^ and high mechanical strength,^10^ the lack of an intrinsic band gap limits their potential applications in electronic devices such as field effect transistors.^6,7^ For this reason, several efforts have been made for finding and developing a wide variety of novel 2D materials, with interesting properties in the fields of nanoscience and nanotechnology.^11−13^

Some good alternatives to graphene are the well-known transition metal dichalcogenides,^14,15^ especially the MoS_2_. Despite its good properties such as a band gap in the visible range,^16,17^ it has been reported that its charge mobilities at low temperature are considerably low, which is something not convenient for applications of high-performance devices.^18^

Also, group 13 and group 15 elements form low dimensional systems on Si and Ge surfaces.^19−21^ Therefore, it is not surprising that elements such as P, As, or Sb can also form stable 2D systems.^22−25^ Among them, a material with outstanding properties and several applications is black phosphorene. It has an intrinsic direct band gap ranging in the visible spectrum,^26^ a strong light-matter interaction,^27^ strong in-plane anisotropy^28,29^ and principally, very high charge carrier mobilities.^30^ These properties make the phosphorene a good candidate for applications such as field effect transistors (FETs),^31,32^ rechargeable batteries,^33^ sensors,^34,35^ and catalysis.^36,37^ Another 2D material based on phosphorus, is blue phosphorene,^22−24^ a wide band gap semiconductor,^38,39^ with promising applications in the field of ultraviolet optoelectronics.^40,41^ Blue phosphorene is also a good candidate for fabrication of gas sensors if it is doped with transition metals.^42^ Previous studies suggest that blue phosphorene can be used in junction-free FETs,^32^ giving rise to the fabrication of transistors of improved characteristics. This last material has been explored mainly from a theoretical point of view, although some experimental works have proved the possibility of its synthesis and preparation.^43^

On the other hand, analog 2D materials based on arsenic with the same atomic structure as phosphorene have been proposed.^22,25^ These compounds are known as buckled arsenene (with a hexagonal structure) and puckered arsenene, with an orthorhombic atomic arrangement. Buckled arsenene is a promising candidate for some applications such as blue-light detectors and LEDs.^44^ Also, if the system is doped with metals, its electronic behavior can be improved suggesting potential applications in spintronic devices.^45^ On the other hand, puckered arsenene shows anisotropic and thickness-dependent semiconductor characteristics,^46^ it also possesses high carrier mobilities,^45,46^ which is a good characteristic for accelerating the electrons during an electrocatalytic reaction.^47^ In contrast to phosphorene, the puckered arsenene possesses a good environmental stability.^48^ Also, its properties make it a good candidate for thermoelectric applications and in the construction of FTEs.^46,49^ Although these materials have been predicted at a theoretical level, many efforts have been made to achieve their experimental synthesis in the near future, because these 2D compounds are considered promising materials for several technological applications.^45−49^

With this in mind, we explored the possibility of building 2D hybrid compounds based on the arsenene and phosphorene structures. Another hybrid 2D structure, siligene, a novel bidimensional compound composed of Si and Ge atoms arranged in a hexagonal lattice,^50^ has been the focus of attention, due to its potential applications for new battery technologies, and electronic components. The hydrogenated siligene has also been shown to be useful for gas detection,^51^ similar to silicon systems.^52^ In this work, we study another hybrid hexagonal system formed by As and P atoms. Additionally, three other possible structures with an orthorhombic arrangement of As and P atoms were also studied.

The existence of these kinds of structures has been predicted previously.^53−58^ However, studies have been focused on some applications of the orthorhombic phase in the form of multilayers, and a deep study concerning a single monolayer is still lacking. Besides, a study regarding the hexagonal structure has not been realized yet, neither from theoretical nor experimental points of view. Although the number of studies on the orthorhombic compound 2D As–P is scarce so far, the results of these works suggest that this compound owns outstanding properties such as tunable band gap, an excellent anisotropic optical response, high specific heat and good ambient stability.^53−58^ In this way, it can be expected the 2D As–P to have several applications such as in anodes of lithium-ion batteries,^53^ energy storage applications,^53^ thin films for usages in optoelectronic,^59^ digital, and radio frequency devices,^59^ infrared photodetectors,^60^ ultrafast photonic devices from near- to mid-infrared regimes,^54^ high-performance field effect transistors,^55^ sensors of toxic gases^57^ and even for biological application for cancer treatments.^61^ By considering the outstanding properties and potential applications of 2D As–P, a deep study at the atomic level is crucial in order to understand the main properties of these structures. For this reason, in the present work, we have developed a first-principles study based on density functional theory, at the atomic level, focused on the structural, electronic, and optical properties of these 2D arsenic–phosphorus (2D As–P) compounds. Finally, previous works have been reported with regard to the prediction of the existence of 2D As–P structures;^62−64^ although these studies are relevant and very useful, a deep study focused on the optical properties, the excitonic effects, and a correct description of the band gap with the GW approach is still lacking. Besides, our study covered more than one possible structure for the system with orthorhombic symmetry, which allowed us to establish the physical properties of each system and determine the most stable configuration.

This paper is organized as follows: in Section 2, we describe in detail the computational methods used to perform our calculations, and Section 3 is devoted to presenting and discussing the obtained results in order to describe the structural, electronic, and optical properties. In this part, we also include a study of the stability of each system. Finally, the conclusions and final remarks are presented in Section 4.

## 2 Computational Methods

All calculations were performed by using the periodic density functional theory as implemented in Vienna Ab initio Simulation Package (VASP) code.^65−67^ The wave function was expanded by employing plane waves with a cutoff kinetic energy equal to 680 eV. The electron–ion was treated by using PAW pseudopotentials.^68^ The monolayers were modeled by using a supercell, consisting of a 1 × 1 periodicity slab and a vacuum space along the *c*-axis of 20 Å in order to avoid the interaction between periodic replicas. The exchange correlation energy was treated within the generalized gradient approximation (GGA)^69^ in the parametrization of Perdew–Burke–Ernzerhof (PBE).^70^ For ground-state calculations, the first Brillouin zone was sampled employing a special *k*-points mesh, in the parametrization of Monkhorst–Pack,^71^ of 21 × 21 × 1 for the hexagonal system, and of 19 × 21 × 1 for the orthorhombic ones. On the other hand, for computation of quasiparticle energies and the absorption spectra, the k-point meshes of 14 × 14 × 1 and 12 × 14 × 1 were used to sample the first Brillouin zones of hexagonal and orthorhombic systems, respectively. The calculations of the electronic band gaps were performed within the G_0_W_0_ approach.^72^ We used 200 empty bands per atom, a vacuum space of 20 Å to avoid interaction among adjacent layers, and a cutoff energy for the response function of 500 eV. The dielectric function imaginary part was obtained by applying the Bethe–Salpeter equation within the Tamm–Dancoff approximation,^73^ for taking into account the electronic effects. We used a cutoff energy of the plane-wave set used to represent the independent-particle susceptibility equal to 400 eV, and a cutoff energy of the plane-wave set used to represent the wave functions to generate the self-energy equal to 600 eV, finally, we used 5 occupied bands and 7 unoccupied bands to compute the optical spectra, which is enough to consider the electronic transitions in an energy range of 0–4.5 eV. The corresponding values of the absorption coefficients were obtained from the real and imaginary parts of the dielectric function.^74^

## 3 Results and Discussion

In this section, we present the results of the computed structural, electronic, and optical properties. We also include the computation of phonon spectra to evaluate the dynamic stability of the structures under study. For testing the mechanical and thermodynamic stabilities, we compute the elastic constants and cohesive energies, respectively.

### 3.1 Structural Properties

We built models of the two-dimensional structures based on both stable phases of arsenene and phosphorene. From this consideration, the hexagonal structure is a possible configuration. On the other hand, it is possible to build three different structures with orthorhombic symmetry.

**Figure 1:**
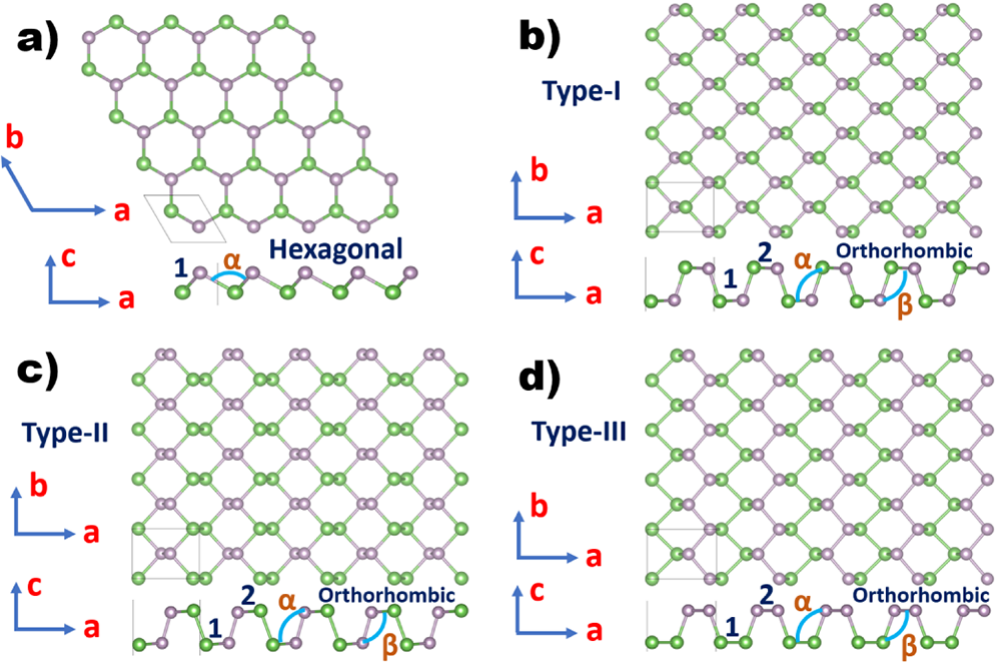
Atomic models of the proposed 2D As–P structures: (a) hexagonal arrangement, (b) orthorhombic type-I, (c) orthorhombic type-II, and (d) orthorhombic type-III. **1** and **2** correspond to bond lengths, and α and β to bond angles.

In Figure 1, we depict the relaxed structures of the suggested models of the two-dimensional As–P systems with the two mentioned symmetries. To achieve the final structures, the atomic positions, as well as the lattice constants were allowed to relax. In Table 1 we show the lattice constants, bond lengths, bond angles, and buckling distances of each relaxed structure, together with the values of the corresponding pristine systems, for comparison purposes.

**Table 1:** Bond Lengths and Angles of 2D As–P Compounds, the Notations are the Corresponding to the Ones of Figure 1

system	ond lengths (Å)	bond angles (deg)
hexagonal	1 = 2.44	α = 95.05
orthorhombic type-I	1 = 2.38; 2 = 2.37	α = 104.1; β = 100.3
orthorhombic type-II	1 = 2.22; 2 = 2.37	α = 103.6; β = 103.6
orthorhombic type-III	1 = 2.38; 2 = 2.26	α = 103.6; β = 102.5

With regard to the hexagonal structure, it can be noticed that the pattern of the blue phosphorene and buckled arsenene is maintained in the hybrid system, and the values of lattice parameters are very close to each other, which means not considerable changes from pristine systems suggesting similar stabilities.

On the other hand, in the three orthorhombic structures (named as type-I, type-II, and type-III) we observe certain differences with respect to the pristine structures. In type-I and type-II structures, the As-atoms are a bit displaced out of the plane of each sublayer, which is not seen in the pristine systems where the atoms of each sublayer lie in the same plane. Conversely, in the last possible structure, named type-III, this effect is not seen, and the same behavior of pristine systems remains. The results of structural properties are summarized in Table 1.

### 3.2 Dynamical Stability

**Figure 2:**
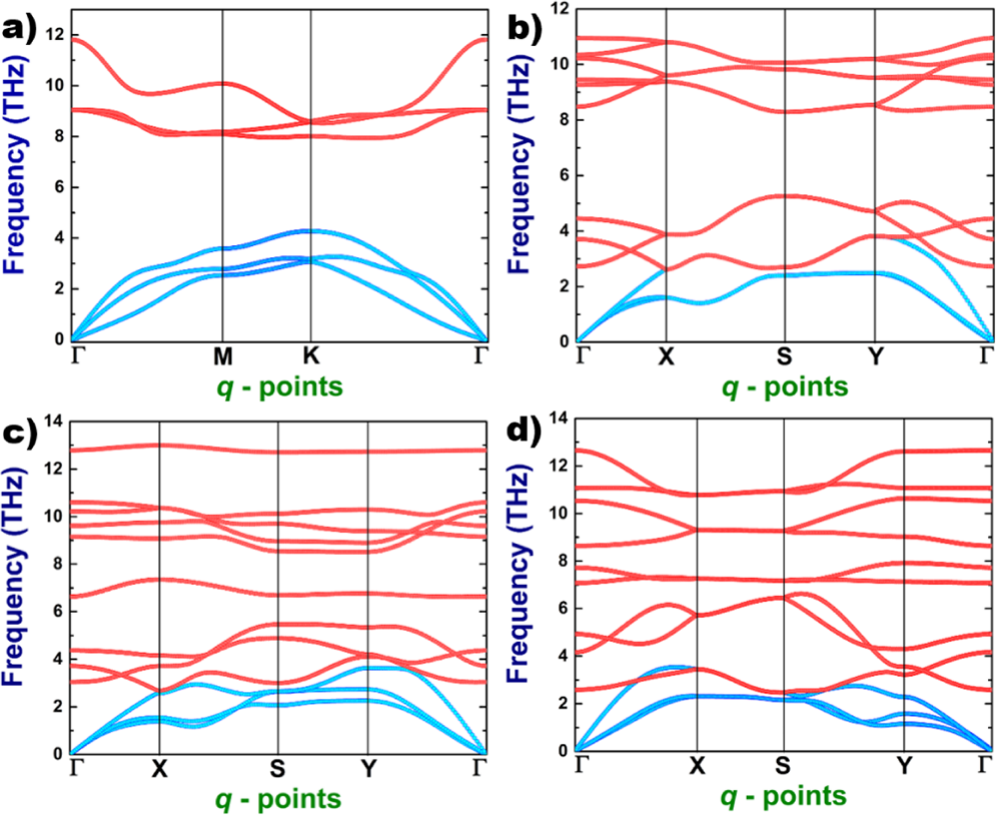
Phonon spectra of 2D As–P compounds: (a) hexagonal, (b) orthorhombic type-I, (c) orthorhombic type-II, and (d) orthorhombic type-III.

In order to evaluate the dynamical stability of each structure, we computed its corresponding phonon spectra, and the results are presented in Figure 2. The criterion of stability was considered to be the existence of positive vibrational frequencies. From Figure 2a, we conclude that the hexagonal structure is dynamically stable because in the entire range of *q*-points we observe just positive vibrational frequencies.

Also, for the orthorhombic systems, the phonon spectra shown in Figure 2b–d reveal that the three types of structures with this kind of symmetry are dynamically stable as we do not observe negative vibrational frequencies in the whole range of *q*-points.

From these results, the dynamical stability has been assured, and it can be expected that the experimental synthesis of these 2D As–P nanostructures can be achieved.

### 3.3 Electronic Properties

**Figure 3:**
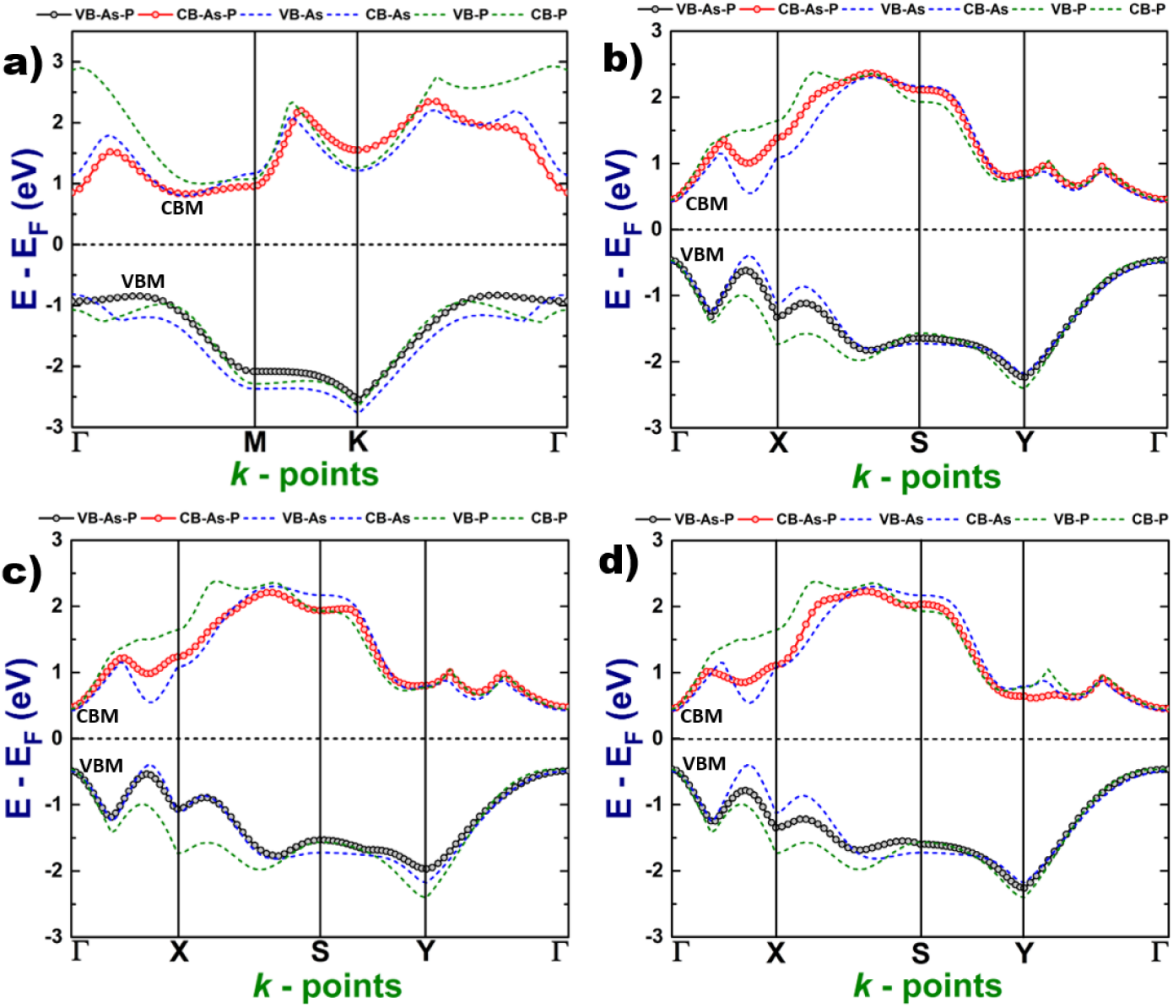
Electronic band structure of the 2D As–P compounds: (a) hexagonal, (b) orthorhombic type-I, (c) orthorhombic type-II, and (d) orthorhombic type-III. Dashed lines refer to electronic bands of pristine systems, these ones were included for comparison purposes.

In order to study the electronic properties of the 2D systems, we have computed the electronic band structures and densities of states. The calculations have revealed that the hexagonal system behaves as an indirect semiconductor; conversely, the orthorhombic ones behave as direct semiconductors. The corresponding band structures are presented in Figure 3.

As part of the study of electronic properties, we computed the band gap within the G_0_W_0_ approach, which allowed us to calculate the quasiparticle energies in order to correct the underestimated values given by standard DFT. In general, the Kohn–Sham band structures are qualitatively correct to describe the excitation energies and provide useful information about the electronic behavior of systems. For this reason, we have computed the electronic band structures and the projected densities of state, in order to describe the main aspects of the electronic behavior of systems under study.

The band structures presented in Figure 3 include a comparison between the bands of 2D As–P systems and those of the corresponding pure systems (arsenene and phosphorene). From this, it can be noticed that, in the hexagonal system, the first conduction band is very similar to that of buckled arsenene, suggesting that the behavior of positive charge carriers (holes) of the hexagonal 2D As–P will be similar to the one of the latter. The first valence band in the region around *M* and *K* high-symmetry points shares some similarities with that of blue phosphorene, indicating that some electronic properties of this material will be seen in the hexagonal 2D As–P. On the other hand, the last valence band shows some aspects that are different from those of the buckled phase of arsenene and phosphorene, suggesting a new set of electronic properties. The main changes can be observed in the regions around the Γ point, where the band is seen to be flat. Two flat regions can be observed in the Γ–*M* and Γ–*K* paths; their presence is favorable for electronic transitions, such as a direct one at the Γ point and another quasi-direct at a *k*-point located in an intermediate point along the Γ–*M*. In general, the last valence band shows the same behavior as that of buckled phosphorene, suggesting that the electrons in hexagonal 2D As–P will have properties similar to those observed in phosphorene. Phosphorene and arsenene pristine buckled systems are direct band semiconductors.

Let us define some useful terms that can be used further. The first conduction band is referred to as the lowest energy conduction band; on the other hand, the last valence band is defined as the highest energy valence band in the band structure. In comparison, the VBM (valence band maximum) and CBM (conduction band minimum) are related to the highest value of energy located in a valence band and the lowest energy value located in a conduction band, respectively. These quantities play a crucial role in determining the electronic behavior of the materials. They are particularly important in semiconductor and insulator materials for determining properties, such as band gap, conductivity, and optical characteristics.

From the band structure of the hexagonal 2D As–P system, we can observe that the VBM is located in an intermediate point along the *K*–Γ path. Conversely, the CBM appears at an intermediate point along the Γ–*M* path. In the same way, the existence of a local maximum in the valence band located along the Γ–*M* path can be noticed; this fact favors the likelihood of having a second indirect transition from this point to CBM, with an energy very similar to the one of the main indirect transition. From the band structure, we can observe that, at the Γ point, there is a local maximum and a local minimum located in the last valence band and first conduction band, respectively, which allows a direct transition with an energy very close to the one of the indirect transition. Because of this, we can consider the hexagonal 2D As–P as a quasi-direct semiconductor.

With respect to the orthorhombic systems, we can notice that, in systems I and type-II, the last valence band follows the same behavior as that in buckled arsenene. It is worth mentioning that, around the Γ-point, the valence band behaves equal to that of black phosphorene; this fact allows us to conclude that the mobilities of valence charge carriers (holes) in the orthorhombic type-I and type-II 2D As–P nanostructures will be the same as the ones of phosphorene. Regarding the first conduction band, we can notice that around the Γ-point, the hybrid system shares the same behavior as that of phosphorene, suggesting that the mobilities will be the same as those observed in this latter material. This is a very important finding, since it has been reported that an outstanding property of phosphorene is related to its high charge carrier mobilities, which makes it a promising candidate for several applications. For this reason, the hybrid material will have the same electronic properties as phosphorene and, consequently, similar potential applications. Let us also mention that in the first conduction band of buckled arsenene we can observe a valley in a point located along the Γ-X path, allowing a direct transition at this point and leading to a high probability of a recombination process (this fact may be inconvenient for certain applications). In this way, an advantage of the hybrid material is related to the fact that the valley in the conduction band (observed in arsenene) appears at a higher value of energy, leading to a low probability that a direct transition at this point to occur. At the same time, this makes the hybrid system behave as a direct band semiconductor, as the VBM and CBM are both located at the Γ point, which is something favorable for optoelectronic applications.

Finally, with respect to the orthorhombic type-III structure, this shows some noteworthy characteristics in its electronic properties. As in the case of the type-II system, the behavior of the last valence and first conduction bands is the same as the one of phosphorene, suggesting similar potential applications as the mobilities of charge carriers will be identical. On the other hand, in the first conduction band, we can notice a flat region around the *Y*-point, which is absent in both arsenene and phosphorene. The existence of this flat region in the hybrid system favors the possibility of indirect transitions from the last valence band at the Γ-point to the first conduction band to some points in the neighborhood of *Y*, or in addition, some intraband transition in the first conduction band.

From the electronic band structures, it is possible to estimate the charge carrier mobilities (electrons and holes), as this information can be obtained from the curvatures of the valence and conduction bands.

The general expression to compute the effective masses of electrons and holes from the band structures is given by eq 1:
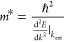
1where m* is the effective mass of the electron or hole, and  is the second derivative of the dispersion curve (electronic band structure). This second derivative is directly related to the curvature of the band. In this way, according to eq 1, the higher the curvature of the band, the lower the effective mass of the charge carrier, i.e., the charge carrier will move faster than the corresponding free particle. For low curvatures of the bands, the opposite behavior is observed. To compute the effective masses of the holes, we consider the curvature of the last valence band, while for the electrons, the one of the first conduction band. Finally, *k*_ext_ refers to the fact that the second derivative of the band is evaluated at the *k*-point at which an extreme value (maximum or minimum) is observed.

The values of the effective masses of the electrons and holes of each system are listed in Table 2. For comparison purposes, we included the results of pristine systems, in order to analyze the effects of adding extra atoms to pristine systems in the behavior of electrons and holes. In general, the mobilities of charge carriers of 2D As–P systems are very similar to those of the pristine systems. In fact, the hybrid systems combine the mobilities of both pristine systems, as it can be noticed from plots of band structures, the dispersion of hybrid and pristine systems are basically the same around the VBM and CBM both located at the Γ point. For this reason, the high mobilities of charge carriers along the *x* and *y* directions are preserved.

**Table 2:** Effective Masses of Electrons and Holes for Pristine and Hybrid 2D As–P Systems^a^

system	effective mass of electrons (in units of *m*_0_)	effective mass of holes (in units of *m*_0_)
b-arsenene	0.1237	0.1186
blue phosphorene	0.1876	0.1360
hexagonal 2D As–P	0.2076	0.2735
p-arsenene	e^*x*^ = 0.1094, e^*y*^ = 0.4230	h^*x*^ = 0.0978, h^*y*^ = 0.5721
black phosphorene	e^*x*^ = 0.1400, e^*y*^ = 1.2300	h^*x*^ = 0.1300, h^*y*^ = 5.3600
type-I orthorhombic 2D As–P	e^*x*^ = 0.0823, e^*y*^ = 0.3887	h^*x*^ = 0.0763, h^*y*^ = 0.6995
type-II orthorhombic 2D As–P	e^*x*^ = 0.0951, e^*y*^ = 0.3812	h^*x*^ = 0.0957, h^*y*^ = 0.6123
type-III orthorhombic 2D As–P	e^*x*^ = 0.0926, e^*y*^ = 0.4123	h^*x*^ = 0.0927, h^*y*^ = 0.8865

a The notation e^*x*^, e^*y*^, h*^x^,* and h^*y*^ refers to the effective mass of electrons along *x*- and *y*-directions.

On the other hand, with basis on the results of the effective masses of electrons and holes, it is possible to compute the charge carrier mobilities. For doing this, we employed the deformation potential theory, proposed by Bardeen and Shockley.^75,76^ From this, it is possible to find the carrier mobility for 2D systems as follows:
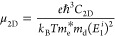
2where *μ*_2D_ is the carrier mobility, *e* is the charge of electron, ℏ is the reduced Planck’s constant, *k*_B_ is the Boltzmann’s constant,  is the effective mass (of electron or hole) along a particular direction (obtained from eq 1), *m*_d_ is the reduced effective mass, which can be computed as , where  and  are the effective masses along *x* and *y* directions respectively. *E*_1_ is defined as the deformation potential constant of the VBM for the hole or CBM for the electron along the transport direction, and can be computed as follows: . In this expression, Δ*E*_*i*_ is the change in the energy of the VBM or CBM as a result of tensile or compressive deformation. This distortion mimics a lattice due to phonons, and it is modeled by multiplying the lattice constant by different factors (0.99, 0.995, 1.005, and 1.01). *l*_0_ is the lattice constant in the direction of transport, and Δ*l* is the change in the lattice constant due to deformation. Finally, *C*_2D_ is the elastic modulus in the propagation direction, and it can be obtained from the expression: , where *E* is the energy of the deformed system, *E*_0_ is the energy of the system at the equilibrium, and *S*_0_ is the area of the 2D lattice at equilibrium.

In this way, we computed the carrier mobilities of electrons and holes of the 2D As–P structures, as well as those of the 2D As and 2D P pristine systems, for comparison purposes. The results are presented in Table 3.

**Table 3:** Charge Carrier Mobilities of Pristine Systems of Arsenene and Phosphorene and of the 2D Systems

system	μ electrons along *x* (cm^2^ V^–1^ s^–1^)	μ holes along *x*(cm^2^ V^–1^ s^–1^)	μ electrons along *y* (cm^2^ V^–1^ s^–1^)	μ holes along *y* (cm^2^ V^–1^ s^–1^)
black phosphorene	24506.13	176.75	425.65	94.59
blue phosphorene	90.41	171.34	90.41	171.34
puckered arsenene	8688.91	166.21	783.12	251.81
buckled arsenene	376.80	203.56	376.80	203.56
hexagonal 2D As–P	1157.11	19259.89	1157.11	19259.89
orthorhombic 2D As–P, type-I	29319.58	911.909	2935.42	1160.88
orthorhombic 2D As–P, type-II	56108.87	682.40	2755.56	1411.47
orthorhombic 2D As–P, type-III	80692.75	757.44	2620.78	1052.04

From the results, it can be noticed that the carrier mobilities are observed to be higher in orthorhombic structures. This property is highly anisotropic, observing a higher mobility along the *x*-direction in all structures. It is worth mentioning that the mobilities improve for 2D As–P structures in comparison with those observed in pristine systems, even for the hexagonal structure. This allows us to conclude that 2D As–P are promising candidates for applications in electronic devices, as the mobilities are even higher than those in phosphorene.

**Figure 4:**
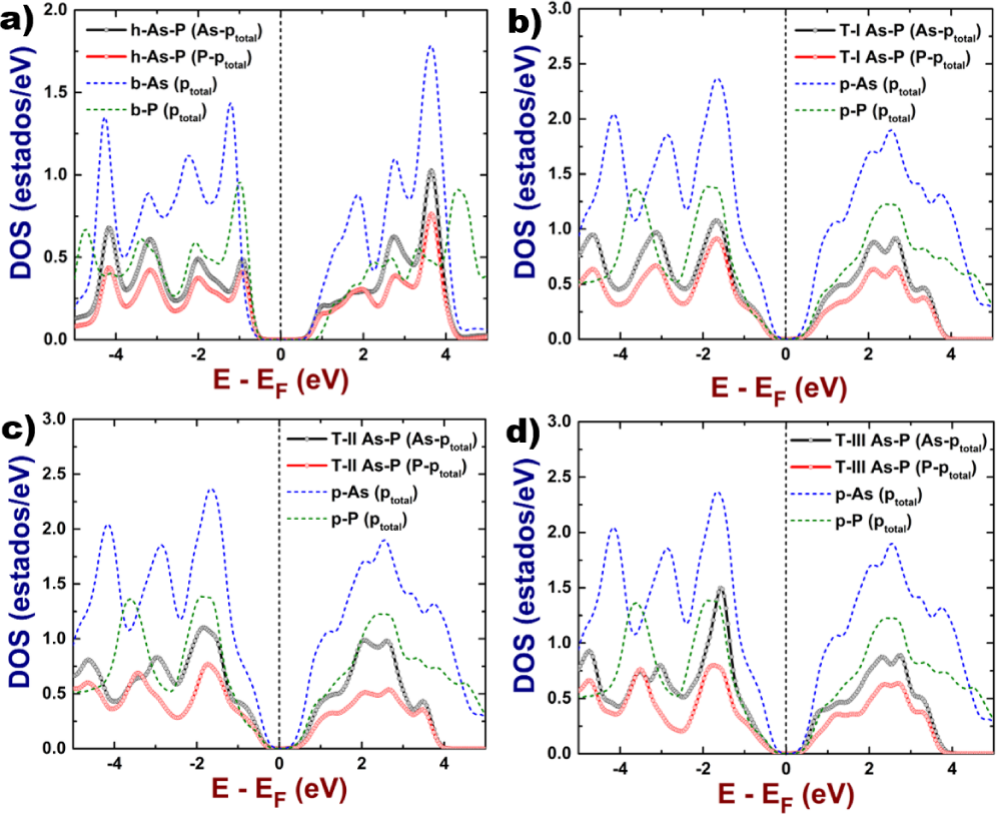
Projected density of states of 2D As–P structures: (a) hexagonal, (b) orthorhombic type-I, (c) orthorhombic type-II, and (d) orthorhombic type-III. Dashed lines are used for PDOS of pristine systems.

Finally, as a part of the study of electronic properties, we have included the calculation of the projected density of states for each system to analyze the contribution of each kind of orbital to the electronic states. The results are presented in Figure 4. These calculations also help us to understand the behavior of the band structures and to establish which orbitals will be involved in the electronic transitions.

Our study is focused on the p-orbitals, as these are the ones that mostly contribute to the electronic states of interest and are involved in the electronic transitions.

The projected density of states of the hexagonal 2D As–P structure shows an overlap of p-orbitals of As and P atoms in the neighborhood of the Fermi level, suggesting that the edges of the last valence and first conduction bands are formed by these kinds of orbitals. As we can notice from the band structure, in the last valence band, besides the absolute maximum, we can observe two additional local maxima which are located in an energy level very close to the one of the absolute maximum (Fermi level). The density of states is useful to explain this special behavior: the arsenic p-orbitals contribute with the states to form the valence band maximum, at energies very close to VBM we observe two local maxima which are formed by the contribution of phosphorus p-orbitals. There is no overlap between the arsenic and phosphorus p-orbitals to generate a single absolute maximum, and this leads to the formation of three different maximum with a very small energy difference among them. We observe the same behavior in the first conduction band, where the CBM and the local minimum located at Γ-point are very close in energies. This can be explained by the density of states: as observed in the valence band, the contribution to electronic states coming from p-orbitals of arsenic and phosphorus are very close one from the other in the region just above the Fermi level, but the overlap is not formed, originating the existence of three different minima.

On the other hand, the orthorhombic type-II structure shows one local maxima in the last valence band besides the VBM, and it can be explained by the density of states, where the p-orbitals of arsenic and phosphorus are very close to one another just below the Fermi level. Despite this, there is no overlap, giving place to the formation of two minima. In the first conduction band, this behavior is not observed. In this case, the edge of the conduction band that originates from the CBM is formed by the contribution of only p-orbitals of arsenic atoms. In the type-III structure, we observe the opposite behavior: the edge of the last valence band that originates the VBM is formed by arsenic p-orbitals, and no other local maximum is observed. Conversely, the p-orbitals of arsenic and phosphorus are very close to one another just above the Fermi level, leading to the formation of a local minimum in the first conduction band around the *Y*-point besides the CBM located at the Γ-point.

As a final part of the study of electronic properties, we computed the electronic band gap within the GW approach to overcome the underestimation in the results when the standard DFT is used. In this way, the GW approximation allows us to obtain trustable results of band gaps, i.e., this approach provides more realistic results in better agreement with the expected values obtained from experiments.

The results of electronic band gaps are presented in Table 4, and we have included the results from standard DFT for comparison purposes. As we can notice, the predicted electronic behavior is the same regardless of the approximation used. Nevertheless, the values obtained from the GW approach are more realistic and it is expected that, after the synthesis of the structures in the near future, the results for band gaps obtained from GW will be in good agreement with the experimental ones.

**Table 4:** Electronic Band Gaps of the Pristine and 2D As–P Systems^a^

system	lectronic band gap (eV)
b-arsenene	2.56 (1.62)
blue phosphorene	3.33 (1.91)
hexagonal 2D As–P	3.36 (1.65)
p-arsenene	1.54 (0.81)
black phosphorene	1.89 (0.90)
type-I orthorhombic 2D As–P	1.80 (0.90)
type-II orthorhombic 2D As–P	1.84 (0.95)
type-III orthorhombic 2D As–P	2.39 (1.12)

a The values in parentheses correspond to those obtained within standard DFT.

The results reveal that the band gap of the hexagonal system lies in the limit of visible spectrum and the near-ultraviolet. Otherwise, the band gaps of the two orthorhombic structures were found to be in the visible spectrum. From this, we can conclude that the hexagonal as well as tetragonal structures are good candidates for optoelectronic applications.

**Figure 5:**
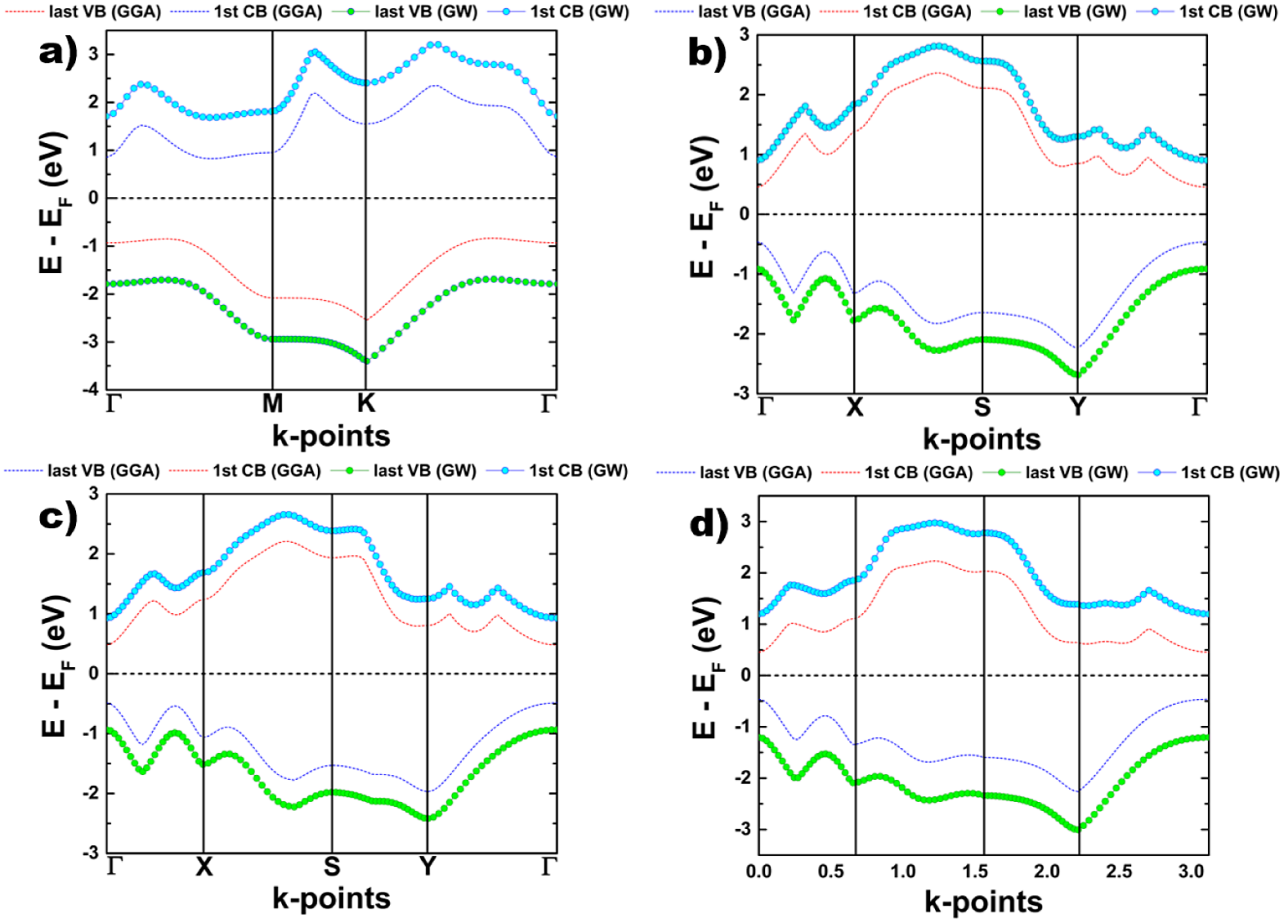
Electronic band structures of the 2D systems: (a) hexagonal 2D As–P, (b) orthorhombic 2D As–P type-I, (c) orthorhombic 2D As–P type-II, and (d) orthorhombic 2D As–P type-III. We plotted the last valence band and the first conduction band.

**Figure 6:**
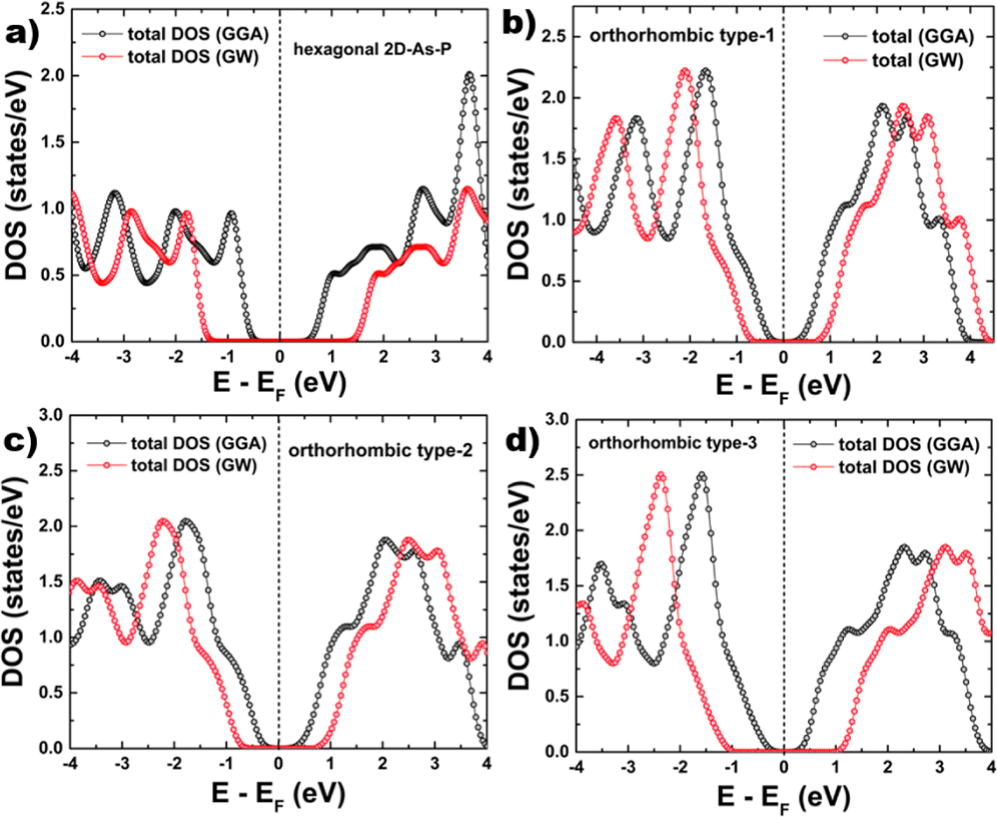
Total density of states of 2D systems: (a) hexagonal 2D As–P, (b) orthorhombic 2D As–P type-I, (c) orthorhombic 2D As–P type-II, and (d) orthorhombic 2D As–P type-III. The calculations were done within the standard-DFT and GW approaches.

For comparison purposes, we depict in Figures 5 and 6 the plots of electronic band structures and the total density of states for each system, computed with the GW approach. As we can notice, the results obtained from the standard DFT are qualitatively correct, and there is no difference with respect to the ones coming from GW calculations. However, the value of the electronic band gap is improved when the GW approach is applied, obtaining better results in agreement with experiments.

### 3.4 Optical Properties

**Figure 7:**
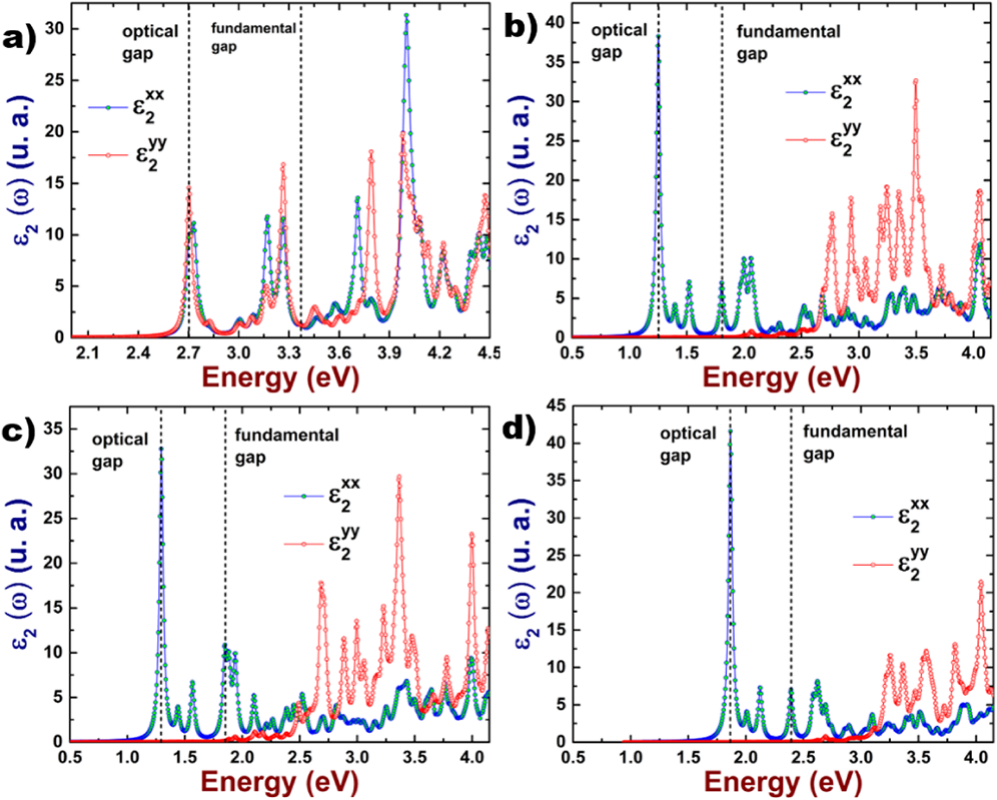
Dielectric function imaginary part of the 2D As–P compounds: (a) hexagonal, (b) orthorhombic type-I, (c) orthorhombic type-II, and (d) orthorhombic type-III.

In order to study the optical properties, we computed the dielectric function imaginary part within the Bethe–Salpeter approximation. This approach takes into account the excitonic effects. In Figure 7 we depict the results of the dielectric function imaginary part of 2D As–P compounds.

The exciton binding energy can be computed from the following expression (eq 3):
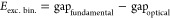
3where gap_fundamental_ refers to the computed value of the electronic band gap, within the G_0_W_0_ approach, on the other hand, gap_optical_ is equal to the energy value at which the first peak in the dielectric function imaginary part appears.

In Table 5, we present the corresponding values of the exciton binding energies; we have included the values of the optical gaps to indicate in which region of the electromagnetic spectrum we can locate the excitonic peaks. We have included the values of pristine systems for comparison purposes.

**Table 5:** Exciton Binding Energies for the 2D As–P Systems^a^

system	exciton binding energy (meV)
b-arsenene	690 (1.87 eV)
blue phosphorene	820 (2.51 eV)
hexagonal 2D As–P	670 (2.69 eV)
p-arsenene	500 (1.04 eV)
black phosphorene	540 (1.35 eV)
type-I orthorhombic 2D As–P	550 (1.25 eV)
type-II orthorhombic 2D As–P	560 (1.28 eV)
type-III orthorhombic 2D As–P	530 (1.86 eV)

a The corresponding values for pristine systems are also included for comparison purposes. The values in parentheses refer to the optical band gaps.

**Figure 8:**
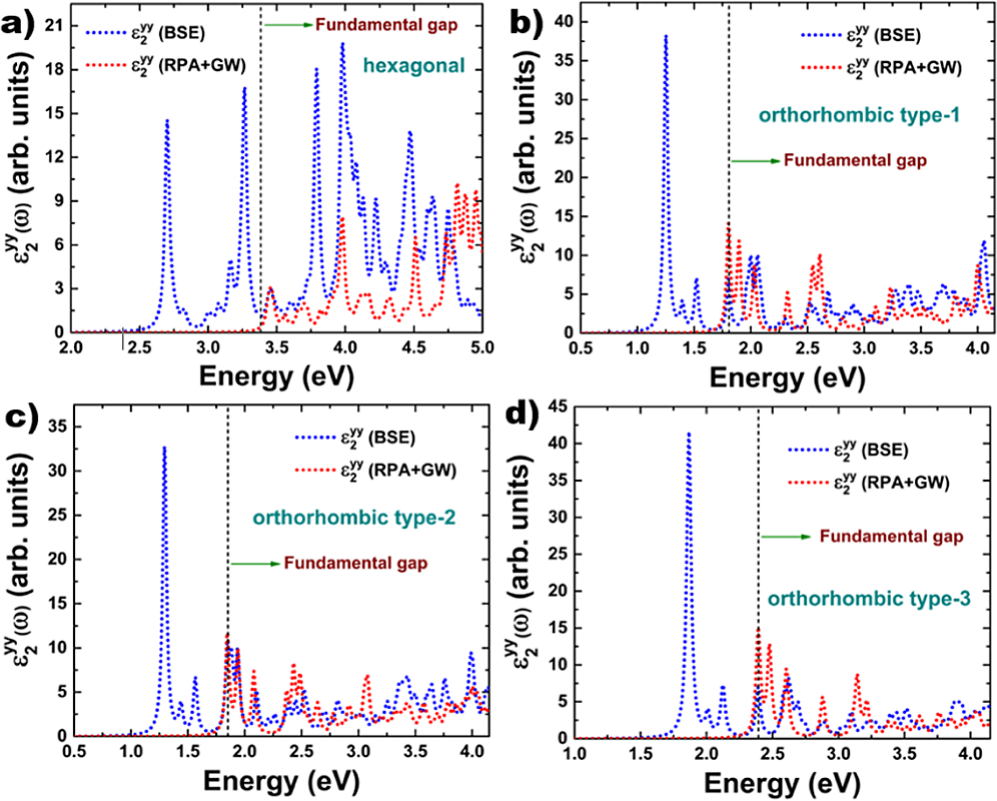
Imaginary part of the dielectric function for 2D systems: (a) hexagonal 2D As–P, (b) orthorhombic 2D As–P type-I, (c) orthorhombic 2D As–P type-II, and (d) orthorhombic 2D As–P type-III. The plots were obtained by applying BSE and RPA+GW approaches.

We can understand the excitonic effects if we compare the plots of the dielectric function imaginary part computed within the BSE and the RPA+GW approaches. In Figure 8 we include the plots of the dielectric function computed by using both approaches. As we can notice, the peaks observed below the band gap are attributed to excitonic effects, which is why there are no peaks present in the plot obtained by using the RPA+GW technique.

**Figure 9:**
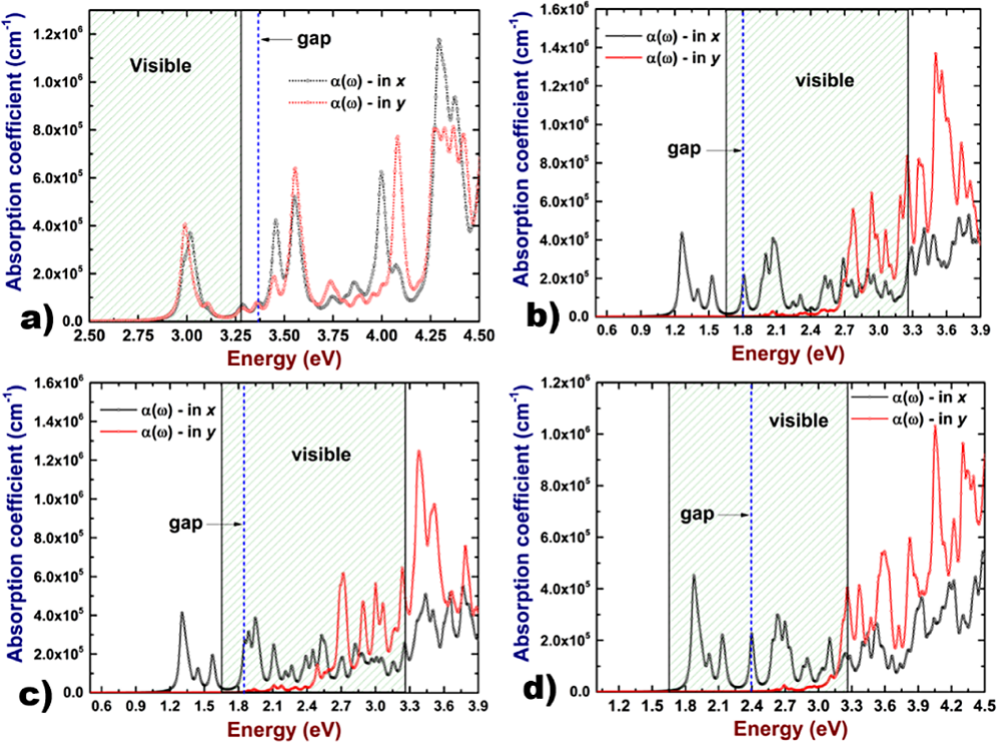
Absorption coefficient of the 2D As–P structure: (a) hexagonal, (b) orthorhombic type-I, (c) orthorhombic type-II, and (d) orthorhombic type-III.

As a final part of the study of optical properties, we computed the absorption coefficient directly from the real and imaginary parts of the dielectric function as follows (eq 4):
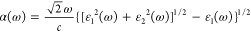
4where ε_1_ and ε_2_ are the real and imaginary parts of the dielectric function respectively, ω is the frequency and *c* is the speed of light in the vacuum. The results can be seen in Figure 9.

Our results reveal that, for the hexagonal 2D As–P system, the absorption is intense in the ultraviolet region and a low absorption is absorbed in the visible range due to excitonic contributions. In this way, the hexagonal system is a good candidate for applications in the field of ultraviolet (UV) optoelectronics. Conversely, in regard to the orthorhombic systems, the electronic band gap lies in the visible range and the light is intensively absorbed in this region; additionally, in the type-I and II systems, we can observe some absorption peaks in the near-infrared region due to excitons. On the other hand, in the type-III system, the absorption peaks including the ones due to excitons lie all in the visible range. This fact makes this nanostructure a promising candidate for optoelectronic applications.

### 3.5 Thermodynamic Stability

For the evaluation of the thermodynamic stability, we computed the corresponding cohesive energies for each system. The cohesive energy can be computed from (eq 5):
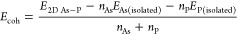
5where *E*_coh_ is the cohesive energy, *E*_2D-As*–*P_ corresponds to the total energy of the 2D As–P structure under consideration, *n*_As_*E*_As(isolated)_ and *n*_P_*E*_P(isolated)_ refers to the number of arsenic/phosphorus atoms in the structure times the energy of an isolated atom of arsenic/phosphorus, respectively, and *n*_As_ + *n*_P_. The criterion of stability is defined by a negative value of the cohesive energy. In Table 6 we have included the cohesive energies of the 2D As–P structures as well as the values of pure arsenene and phosphorene systems. The results reveal that the 2D As–P compounds are thermodynamically stable, with cohesive energies very close to the ones of their pristine counterparts (arsenene and phosphorene), indicating similar stabilities.

**Table 6:** Cohesive Energies for 2D As–P Compounds^a^

system	
b-arsenene	–3.480^22^
p-arsenene	–3.481^22^
blue phosphorene	–3.480^22^
black phosphorene	–3.481^22^
hexagonal	–3.130
orthorhombic type-I	–3.153
orthorhombic type-II	–3.172
orthorhombic type-III	–3.154

a We have included the results for pristine systems for comparison purposes.

In addition, we computed the formation energies per atom of each 2D As–P system, and besides, we computed the corresponding values for the pristine 2D As/P structures, for comparison purposes. In this way, the formation energies can be obtained as follows (eq 6):
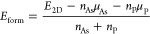
6where *E*_form_ is the formation energy, *E*_2D_ is the total energy of the 2D As–P or the pristine 2D As/P structure, *n*_As_ is the total number of As atoms, *n*_P_ is the total number of P atoms, *μ*_As_ is the chemical potential of arsenic, which is defined as the total energy per atom of bulk phosphorus, and *μ*_P_ is the chemical potential of phosphorus, and can be defined as the total energy per atom of bulk arsenic.

By applying eq 6, we can obtain the formation energies per atom for each structure; the results are presented in Table 7.

**Table 7:** Formation Energies per Atom for Each 2D As–P System and the Pristine Arsenene and Phosphorene Structures

system	
blue phosphorene	0.0361
black phosphorene	0.0347
buckled arsenene	0.2435
puckered arsenene	0.2806
hexagonal 2D As–P	0.2268
orthorhombic 2D As–P type-I	0.2040
orthorhombic 2D As–P type-II	0.1849
orthorhombic 2D As–P type-III	0.2031

From the results, it can be noticed that the phosphorene structures have the lowest formation energies, suggesting that they are the most favorable to be formed from the bulk systems. On the other hand, the presence of arsenic atoms makes the formation energies increase; however, these values are small enough to be favorable to be formed.

### 3.6 Mechanical Stability

For assessing the mechanical stability, we computed the independent elastic constants of each system. After this, we evaluated the sufficient and necessary conditions (stated in ref (77)) for mechanical stability based on the obtained values of elastic constants.

In Table 8, we include the values of the independent elastic constants of each structure under study.

**Table 8:** Computed Elastic Constants of the 2D As–P Structures

elastic constant (GPa)	exagonal			
C11	22.734	9.247	9.026	9.807
C22	C11	39.406	38.027	40.322
C33	0.345	0.445	0.200	0.709
C44	0.354	0.399	0.218	0.222
C55	C44	0.034	0.010	0.062
C66	9.605	9.320	9.290	8.201
C12	3.525	8.999	8.431	8.283
C13	0.037	0.338	0.136	0.394
C23	C13	0.130	–0.167	0.485

In this way, for the hexagonal structure, the necessary and sufficient conditions for assessing the mechanical stability are as follows (eqs 7–9):
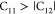
7
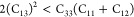
8
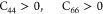
9

On the other hand, the conditions for ensuring mechanical stability of orthorhombic type-II and type-III structures are given by eqs 10–12:12
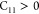
10
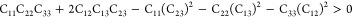
11
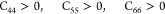
12

By using eqs 7 to 12 with the obtained values of elastic constants presented in Table 8, it can be noticed that all the conditions are fulfilled for the hexagonal and type-I, type-II, and type-III orthorhombic systems, which allows us to conclude that all the structures are mechanically stable.

**Figure 10:**
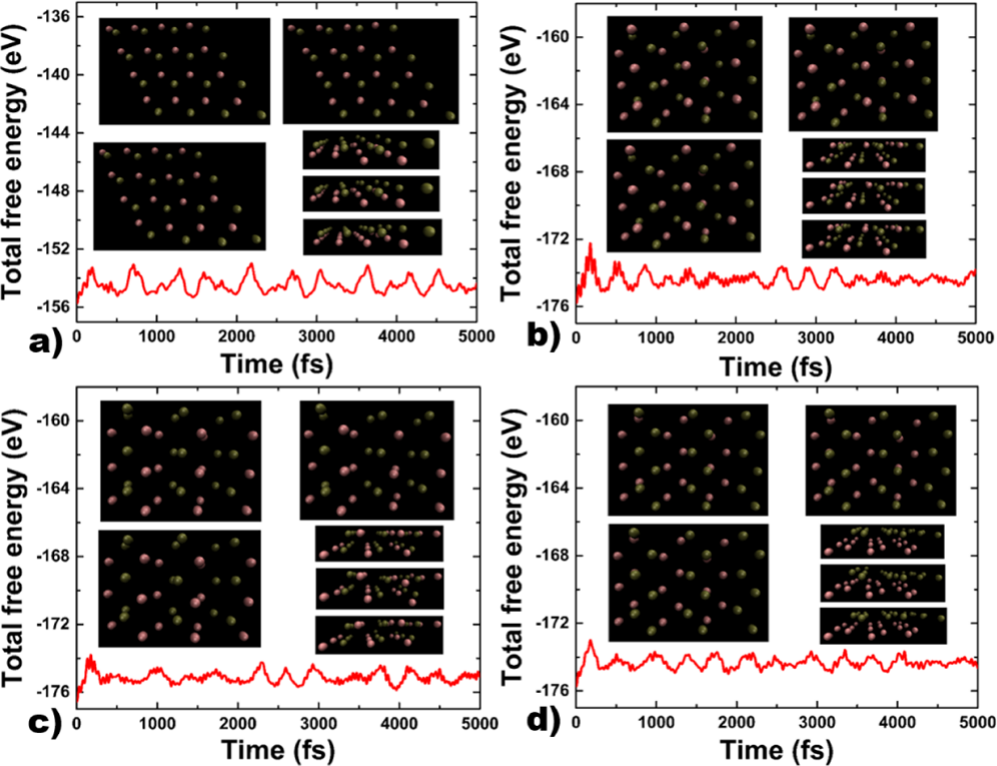
Results of ab initio molecular dynamics calculations for (a) hexagonal 2D As–P, (b) orthorhombic 2D As–P type-I, (c) orthorhombic 2D As–P type-II, and (d) orthorhombic 2D As–P type-III.

Finally, we performed ab initio molecular dynamics calculations in order to assess the stability of the systems at room temperature. The results are depicted in Figure 10; we have plotted the evolution of the energy as a function of time. We can notice small variations in energy during the considered period of time. Besides, we have included some representative figures depicting the evolution over time of the structures. From the results, it is possible to conclude that the structures are not broken at room temperature; we just observe very small distortions, but the bonds are not broken and the structure is preserved.

## 4 Conclusions

We have performed a first-principles study about the structural, electronic, and optical properties of 2D As–P compounds. The study considered one system with a hexagonal structure and three possible structures with orthorhombic symmetry. The structural properties suggest that the atomic arrangements of the systems are very similar to those of their pristine counterparts, suggesting no considerable distortions of unit cells and atomic positions. The computation of phonon spectra reveals that all of the structures are dynamically stable, as no negative frequencies were observed in the whole range of *q*-points. The calculation of cohesive energies ensured the thermodynamic stability of all compounds. In the same way, the mechanical stability for all of the systems was guaranteed, as the sufficient and necessary conditions for the elastic constants were fulfilled. Besides, we assessed the stability at room temperature (300 K) according to our results of ab initio molecular dynamics. From electronic band structures, it is possible to conclude that the hexagonal compound behaves as an indirect band gap semiconductor, and conversely, the orthorhombic structures behave as direct band gap semiconductors. The electronic band structure also reveals that high charge carrier mobilities are observed in all of the compounds. We also computed the charge carrier mobilities, which verifies that the mobilities of electrons and holes are improved with respect to the pristine 2D As/P systems. On the other hand, as a part of the study of electronic properties, we computed the electronic band gap within the G_0_W_0_ approach, to obtain more realistic values of band gaps which are underestimated in the standard DFT calculations. From the results, we found that the hexagonal structure is a wide band gap semiconductor, with a value lying in the near-ultraviolet. On the other hand, the orthorhombic structures have band gaps lying in the visible range of the electromagnetic spectrum. From the study of optical properties, we found large values for exciton binding energies, such as those observed in other 2D materials, suggesting that 2D As–P compounds are very stable after light absorption, avoiding recombination processes. The absorption coefficients show that the hexagonal structure absorbs light in the range of near UV and some absorption is observed in the visible region due to excitons. Finally, the orthorhombic compounds absorb light in the visible range with some contributions in the near-infrared as a result of excitonic contributions. From our results, we can conclude that the 2D As–P compounds are promising candidates for optoelectronic applications.
